# Optimal carbon dioxide insufflation pressure during robot-assisted thyroidectomy in patients with various benign and malignant thyroid diseases

**DOI:** 10.1186/1477-7819-10-202

**Published:** 2012-09-27

**Authors:** Hoon Yub Kim, Yoon Ji Choi, Hae-Na Yu, Seung Zhoo Yoon

**Affiliations:** 1 Department of Surgery, College of Medicine, Korea University, Seoul, South Korea; 2 Department of Anesthesiology and Pain Medicine, Asan Medical Center, University of Ulsan, Seoul, South Korea; 3 Department of Anesthesiology and Pain Medicine, College of Medicine, Korea University, Seoul, South Korea

**Keywords:** Benign and malignant thyroid diseases, Bilateral axillo-breast approach, Da Vinci, Carbon dioxide insufflation, Pressure, Robot-assisted thyroid surgery

## Abstract

**Background:**

Currently, data are not available concerning a safe insufflation pressure that provides a proper view of the surgical field without adverse metabolic and hemodynamic changes in humans undergoing the robot-assisted thyroidectomy bilateral axillo-breast approach (BABA) using the da Vinci robotic surgical system. The purpose of this study was to determine the optimal carbon dioxide (CO_2_) insufflation pressure in patients with various benign and malignant thyroid diseases when using the da Vinci robotic surgical system.

**Methods:**

A total of 32 patients underwent thyroid surgery at 6 (n = 15), 9 (n = 15), and 12 (n = 2) mmHg. The partial pressure of carbon dioxide (PaCO_2_), pH, cardiac output, heart rate, and mean arterial pressure were measured at baseline, 30 min and 1, 1.5, and 2 hours after CO_2_ insufflation, and 30 min after desufflation.

**Results:**

CO_2_ insufflation of 12 mmHg caused severe facial subcutaneous emphysema, hypercarbia, and acidosis during robot-assisted thyroidectomy with BABA. The study was stopped before completion for the patients’ safety in accordance with the study protocol. Applying 6- or 9- mmHg of CO_2_ insufflation pressure caused increases in PaCO_2_ and decreases in arterial pH. However, vital signs were stable and pH and PaCO_2_ were within the physiologic range during the surgery in the 6- and 9-mmHg groups.

**Conclusions:**

We propose that a CO_2_ insufflation pressure under 10 mmHg in robot-assisted thyroidectomy with BABA is the optimal insufflation pressure for patient safety.

## Background

Since endoscopic neck surgery was introduced into clinical practice in 1995 by Gagner [1], robot-assisted thyroidectomy via the bilateral axillo-breast approach (BABA) using the da Vinci robotic surgical system has been successfully used for various benign and malignant thyroid diseases with a low rate of adverse effects and excellent cosmetic outcomes [2,3]. BABA is performed by using two circumareolar ports and two axillary ports, which is useful for identifying anatomy and dissection during surgery [3,4]. The endo-wrist function of the instrument in the da Vinci robotic surgical system makes it possible to employ complex techniques, even in difficult areas with limited access.

During laparoscopic surgery, carbon dioxide (CO_2_) gas is usually insufflated into the body cavity, and an electronic variable-flow insufflator controls the insufflation pressure [5]. A higher CO_2_ insufflation pressure allows a better view of the surgical field. However, because the insufflation pressure can affect the severity of hypercarbia, balancing the insufflation pressure between a better view of the surgical field and preventing hypercarbia is very important. In laparoscopic surgery, the insufflation pressure should be maintained below 16 mmHg in order to prevent significant hypercarbia [5,6].

The use of CO_2_ insufflation in the neck potentially causes adverse metabolic and hemodynamic changes as well as tachycardia and massive subcutaneous emphysema [7]. According to Bellantone *et al*., hypercarbia, moderate acidosis, and a slight increase in mean arterial pressure occurred in pigs undergoing endoscopic neck surgery at 15 mmHg [8]. They proposed that CO_2_ neck insufflation is safe at 10 mmHg. Therefore, during endoscopic neck surgery, the authors empirically used 6 mmHg of CO_2_ as the maximal CO_2_ insufflation pressure in BABA endoscopic thyroidectomy [3,4]. However, concrete data supporting the rationale of using 6 mmHg of CO_2_ insufflation pressure in endoscopic thyroidectomy with BABA have not been published. In addition, no data are currently available concerning a safe insufflation pressure that can provide a proper view of the surgical field without adverse metabolic and hemodynamic changes in humans undergoing robot-assisted thyroidectomy with BABA by using the da Vinci robotic surgical system (RaBABA).

Therefore, the purpose of the present study was to determine the optimal CO_2_ insufflation pressure in patients undergoing RaBABA.

## Methods

After obtaining approval from the institutional review board and written informed consent, 32 patients with American Society of Anesthesiologists (ASA) physical status I–II undergoing elective RaBABA were randomly assigned to the three groups described below. The ages of the patients ranged from 18 to 60 years.

Patients were randomly assigned to one of the three groups. The groups were named on the basis of the CO_2_ gas pressure used to insufflate the peritoneal cavity. Patients with previous neck or breast surgery, severe cardiopulmonary disease, morbid obesity, and neck or vertebral abnormality were excluded from this study.

The patients were premedicated with midazolam (2 mg) and glycopyrrolate (0.2 mg) intramuscularly 30 min before anesthesia. Standard monitoring included electrocardiogram, noninvasive arterial blood pressure, pulse oximetry, end-tidal CO_2_, bispectral index, and expiratory gas concentration. Anesthesia was induced with intravenous propofol and was maintained with 50% nitrous oxide in oxygen and an end-tidal concentration of 2 to 3 vol% of sevoflurane. Intravenous rocuronium bromide was used to facilitate tracheal intubation. The radial artery was cannulated with a 20-gauge catheter to monitor arterial blood pressure. The Vigileo^TM^ System (FloTrac, Edwards Lifesciences, Irvine, CA, USA) was connected to the arterial line to monitor the cardiac index (CI) and cardiac output (CO). Mechanical ventilation was maintained with oxygen with air (FiO_2_ 0.5) at a constant tidal volume (8 mL/kg) and frequency (12 breaths/min) during the study. Lactated Ringer’s solution was used for volume replacement during surgery.

If any one of the termination criteria was met during the surgery, insufflation pressure was reduced to below 5 mmHg. If the study was terminated in two consecutive cases within the same group, the study group was excluded and a new randomization code was generated between the remaining groups. The termination criteria of the study were as follows: (1) persistent acidosis (pH ≤7.15) or hypercarbia (partial pressure of carbon dioxide (PaCO_2_) ≥50 mmHg) for 30 min; (2) the mean blood pressure decreased to below 50 mmHg; (3) the CI decreased to below 2 L/min; (4) the heart rate increased to over 110 beat/min; and (5) the occurrence of serious adverse events, such as subcutaneous emphysemas, as determined by the attending anesthesiologists.

All operations were performed by a single surgical team. According to previous reports [3,4], the patient was prepared in the supine position with a pillow placed under the shoulders to maintain neck extension with both arms naturally abducted on both sides. After the vital signs were stabilized, base line blood gases and hemodynamic parameters were recorded. The flaps were raised using a vascular tunneler, and bilateral axillary and two circumareolar 8 mm to 12 mm ports were inserted. The flap extended from the superior border of the thyroid cartilage to the 4-cm inferior area under the clavicle and laterally from just beyond the lateral margin of sternocleidomastoid. At this point, CO_2_ insufflation was performed, the pressure of CO_2_ insufflation was determined by the designated group, and the time 0 of insufflation was recorded. The working space was maintained by continuous CO_2_ insufflation pressure. Total thyroidectomy via the BABA approach using the da Vinci robot system was performed using the general methods [3,4].

Heart rate, mean arterial pressure (MAP), pH, PaCO_2_ (arterial), CI and CO were measured at baseline, 0 min, 30 min, 1 hour, 1.5 hours, and 2 hours after CO_2_ insufflation and desufflation, and at 30 min after desufflation.

Statistical analysis was conducted using SigmaStat 3.5 for Windows (Systat Software, Inc., Chicago, IL, USA). Variables between groups were compared using the two-sample *t*-test or Mann–Whitney rank sum test. Variables at each time point within a group were compared using repeated measures analysis of variance. A *P* value <0.05 was considered statistically significant.

## Results and discussion

### Enrollment, group assignment, and analysis of patients

In total, 32 patients were included in this study. These patients were randomly allocated to three groups: the 6-mmHg group (n = 15), the 9-mmHg group (n = 15), and the 12-mmHg group (n = 2). Severe facial subcutaneous emphysema was observed at 15 and 30 minutes after the initiation of CO_2_ insufflation in the two patients in whom the surgery was conducted using a CO_2_ insufflation pressure of 12 mmHg. Furthermore, rapid progression of hypercarbia and acidosis was observed. The study was stopped midway in accordance with the termination criteria. In addition, the 12-mmHg group was dropped because two consecutive cases developed severe facial subcutaneous emphysemas, hypercarbia, and acidosis. The remaining patients in the 12-mmHg group were randomly allocated to the other two groups and successfully participated in this study. Statistically significant differences were not observed in any of the variables, including neck circumstance before and after CO_2_ insufflation, between the two groups (Table 1).

**Table 1:** **Demographic characteristics and baselinedata**

	**6 mmHg**	**9 mmHg**
Age (years)	46.8 ± 8.4	36.0 ± 6.7
Weight (kg)	58.4 ± 6.8	58.5 ± 6.8
Height	158.9 ± 5.1	160.0 ± 4.0
Volume of CO_2_ insufflation (L)	1,390.6 ± 417.3	1,662.3 ± 553.3
Duration of CO_2_ insufflation (min)	240.3 ± 48.8	220.6 ± 49.9
Neck circumstance before CO_2_ insufflation	16.0 ± 2.8	15.8 ± 1.3
Neck circumstanceafter CO_2_ insufflation	16.8 ± 3.3	16.7 ± 1.3

Data are expressed as the mean ± SD. Statistically significant differences in the variables were not observed between the two groups.

### Analysis of hemodynamic parameters

**Figure 1:**
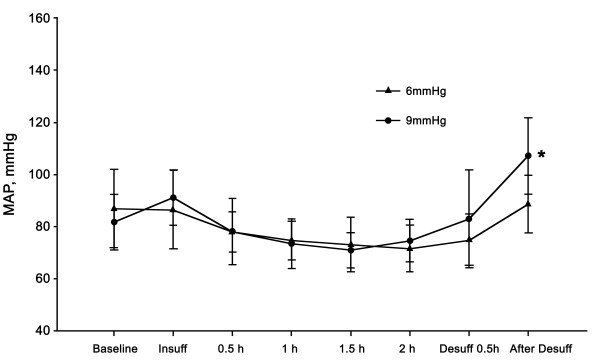
**Changes in mean arterialpressure (MAP).** A slight increase in MAP at 30 min after desufflation compared to the baseline (*P* <0.05) was observed in the 9-mmHg group. Significant differences were not observed between the groups. Data are expressed as the mean ± SD. * *P* <0.05 versus baseline value.

**Figure 2:**
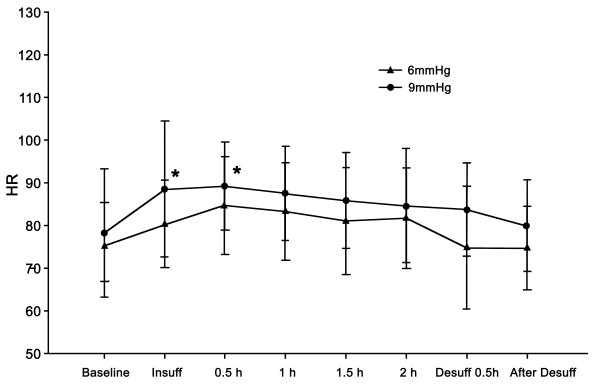
**Changes in heart rate(HR).** Although a slight increasing trend was observed in HR over time, this change was tolerable. A slight increase in HR at insufflation and 30 min after insufflation compared to the baseline (*P* <0.05) was observed in the 9-mmHg group. Significant differences were not observed between the groups. Data are expressed as the mean ± SD. * *P* < 0.05 versus baseline value.

**Figure 3:**
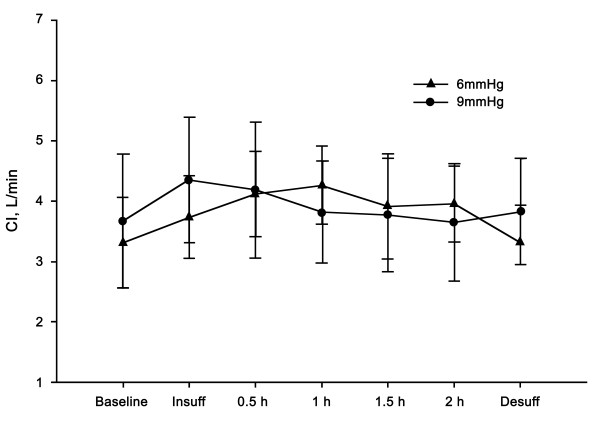
**Changes in cardiac output(CO).** Changes in CO were tolerable even at 9 mmHg of insufflation. Significant differences were not observed between the groups. Data are expressed as the mean ± SD.

Differences in MAP, heart rate (HR), CO, and CI were not observed between the groups at any of the time points. After desufflation, the MAP was increased compared to the baseline value in the 9-mmHg group (*P* <0.05) (Figure 1). The HR of the 9-mmHg group was increased compared to the baseline value at 30 min after insufflation, but returned to the baseline level after 1 hour (Figure 2). The CO and CI did not show statistically significant differences compared to the baseline values at any of the time points (Figure 3).

### Analysis of metabolic parameters

**Figure 4:**
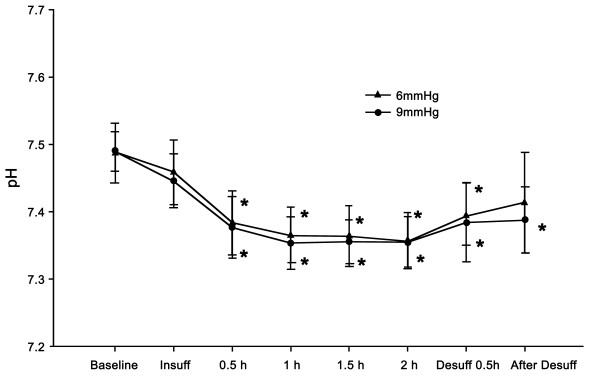
**Changes in pH.** Decreasing pH was observed at 30 min and 1, 1.5, and 2 hours after insufflation and desufflation compared to the baseline in the 6-mmHg group (*P* <0.05). After desufflation, pH levels returned to the baseline at 30 min in the 6-mmHg group. Decreasing pH was observed at 30 min and at 1, 1.5, and 2 hours after CO_2_ insufflation and desufflation, and at 30 min after desufflation compared with the baseline in the 9-mmHg group. Significant differences were not observed between the groups. Data are expressed as the mean ± SD. * *P* <0.05 versus baseline value.

**Figure 5:**
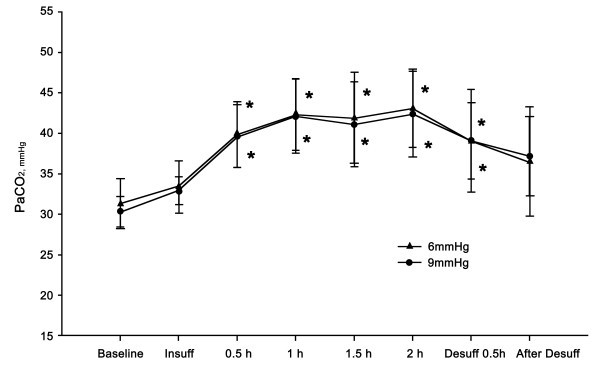
**Changes in PaCO**_**2**_**.** Increasing PaCO_2_ was observed at 30 min and 1, 1.5, and 2 hours after insufflation and at desufflation compared with the baseline in the 6- and 9-mmHg groups (*P* <0.05). At 30 min after desufflation, PaCO_2_ levels returned to the baseline in the 6- and 9-mmHg groups Significant differences were not observed between the groups. Data are expressed as the mean ± SD. * *P* <0.05 versus baseline value.

Patients undergoing surgery did not develop acidosis or hypercarbia in the 6- or 9-mmHg groups (Figures 4, 5). Changes in pH and PaCO_2_ were within the physiologic range at each time point. In addition, no differences in pH and PaCO_2_ were observed between the groups at any of the time points. In the 6-mmHg group, decreased pH and increased PaCO_2_ compared to the baseline value was observed at 30 min and at 1, 1.5, and 2 hours after insufflation. The pH and PaCO_2_ levels returned to the baseline value at 30 min after desufflation. In the 9-mmHg group, decreased pH and increased PaCO_2_ compared to baseline was observed at 30 min and 1, 1.5, and 2 hours after insufflation. PaCO_2_ levels returned to the baseline value at 30 min after desufflation. However, the pH was decreased until 30 min after desufflation.

Our results showed that a CO_2_ insufflation pressure of 12 mmHg caused severe facial subcutaneous emphysema, hypercarbia, and acidosis during RaBABA. Although applying 6- or 9-mmHg of CO_2_ insufflation pressure increased PaCO_2_ and decreased arterial pH, the vital signs were stable and pH and PaCO_2_ were within the physiologic range during surgery.

Subcutaneous emphysema is a frequent complication during endoscopic surgery [5,9]. Subcutaneous emphysema aggravates hypercarbia and acidosis because of an increase in the total gas exchange area [10]. Gottlieb *et al*. [7] reported that massive subcutaneous emphysema, hypercarbia, supraventricular tachycardia, and acidosis occurred in a patient undergoing endoscopic trans-cervical parathyroidectomy at a CO_2_ insufflation pressure of 20 mmHg. According to Bellantone *et al*., hypercarbia, moderate acidosis, and a slight increase in mean arterial pressure occurred in pigs undergoing endoscopic neck surgery at 15 mmHg [7]. In addition, a CO_2_ insufflation of 15 mmHg caused CO_2_ accumulation and acute hypercarbia, which can lead to intracranial angiectasis, increased brain blood volume, and subsequent intracranial hypertension [11,12]. Therefore, the severe facial subcutaneous emphysema experienced by the two patients in the 12-mmHg group was an anticipated event before the collection of study data. However, because several previous studies [7,8] suggested that using an insufflation pressure of 10 to 15 mmHg was safe in endoscopic neck surgery in an animal model, we hoped that 12 mmHg would provide a better view of the surgical field without adverse metabolic and hemodynamic changes.

From the surgeon’s point of view, 9 mmHg pressure of CO_2_ insufflation may give us several advantages over 6 mmHg pressure of insufflation when performing RaBABA. First, 9 mmHg insufflation provides wider operative space than 6 mmHg insufflation. Unlike the case with non-articulating linearly moving instruments used in conventional endoscopic thyroidectomy, various kinds of articulating instruments which enable almost 360 degrees of freedom of movement are applied through the robotic arms during robotic thyroidectomy, and to use the articulating instruments effectively a wider operative space is needed than is in conventional endoscopic thyroidectomy. In other words, higher flap and wider operative space are necessary to fully take advantage of robotic arm movements during RaBABA. This wider space may be achieved by the maximal safe CO_2_ insufflation pressure which turned out to be 9 mmHg in this study. Second, a higher raised flap in higher CO2 insufflation pressure helps to keep the surgeon's view clear for a longer time and lessens the need to cleanse the lens of the endoscopic camera during RaBABA. The surgeons performing RaBABA are hindered by the visual disturbance that results from the contamination of the endoscopic camera lens by subcutaneous and subplatysmal fat tissues, especially in obese patients. A more highly elevated flap in 9 mmHg CO_2_ insufflation pressure than in 6 mmHg helps to keep the camera view clear for a longer operative time in these obese patients undergoing RaBABA. Third, in turn, higher insufflation pressure can be somewhat helpful in diminishing the total operation time of RaBABA, especially in obese patients. However, in our study, there was no statistically significant difference in total operation time between the 6 mmHg and 9 mmHg groups (240.3 min in the 6 mmHg group versus 220.6 min in the 9 mmHg group), which might be a result of the limited numbers of enrolled patients in the two groups and also the lack of morbidly obese patients in either group.

Because CO_2_ has a high solubility, the absorption dose of CO_2_ is proportional to CO_2_ pressure and the duration of insufflation [13]. CO_2_ gas is easily diffused in loose areolar and rough tissue [14], such as in the neck. In previous studies performed in a porcine model, PaCO_2_ increased and pH decreased slightly without acidosis or adverse hemodynamic changes in the 10-mmHg group [8,15]. In our study, arterial CO_2_ and pH were changed after CO_2_ insufflation in the 6- and 9-mmHg groups. The increased PaCO_2_ caused the decrease in pH. However, the changes in pH and PaCO_2_ were within the physiologic range in the 6- and 9-mmHg groups during the insufflation period. Hypercarbia due to CO_2_ insufflation can cause epinephrine and norepinephrine release, resulting in hypertension and tachycardia [16]. However, the blood pressure (BP) response to hypercarbia varies and cannot always be used as a diagnostic sign [16,17]. In addition, although tachycardia commonly occurs with hypercarbia during CO_2_ insufflation [5,6,16], previous studies revealed that changes in HR were inconsistent [17,18]. In our study, the BP did not change during the insufflation period but the HR was slightly increased immediately after insufflation. However, the change in HR was tolerable and self-limited.

This study had several limitations. Our results showed that the CO was not changed in the 6- or 9-mmHg groups compared to the baseline value during the study period. The continuous thermodilution technique using a catheter placed in the pulmonary artery is widely used to assess CO in critically ill patients [19,20]. Although this method is believed to be quite accurate under most clinical conditions [21,22], the process of acquiring central venous access and a balloon floating through the right heart can cause complications, which are sometimes fatal [23,24]. The Vigileo/FloTrac is a valuable tool for the management of patients with diseases such as cardiovascular dysfunction and critical illness or in those undergoing major surgery [25,26] or who are exposed to situations of changing arterial pulse contour [27-29]. Thus, we used the Vigileo/FloTrac to measure CO. A study that used the thermodilution technique with a Swan − Ganz catheter in a porcine model reported that CO increased in the 10-, 15-, and 20-mmHg groups, but these changes were not statistically significant; these results are quite similar to our findings herein.

## Conclusions

In conclusion, we propose that a CO_2_ insufflation pressure below 10 mmHg in robot-assisted thyroidectomy with BABA is an optimal insufflation pressure.

## Abbreviations

BP: blood pressure; BABA: bilateral axillo-breast approach; CI: cardiac index; CO: cardiac output; HR: heart rate; MAP: mean arterial pressure; RaBABA: robotic bilateral axillo-breast approach.

## Competing interests

The authors declare that they have no competing interests.

## Authors’ contributions

HYK helped conduct the study and write the manuscript, YJC helped analyze the data and write the manuscript, HNY writed the manuscript and SZY helped design the study, conduct the study, analyze the data, and write the manuscript. All authors read and approved the final manuscript.
